# Palliative radiotherapy utilization for cancer patients at end of life in British Columbia: retrospective cohort study

**DOI:** 10.1186/1472-684X-13-49

**Published:** 2014-11-18

**Authors:** Jin Huang, Elaine S Wai, Francis Lau, Paul A Blood

**Affiliations:** School of Health Information Science, University of Victoria, PO BOX 1700 STN CSC, Victoria, British Columbia Canada; Department of Surgery, Faculty of Medicine, University of British Columbia, 950 West 10th. Avenue, Vancouver, BC Canada; Division of Radiation Oncology, BC Cancer Agency, Vancouver Island Centre, 2nd Floor, 2410 Lee Avenue, Victoria, BC Canada

**Keywords:** Palliative radiotherapy, Radiation therapy, End of life care, End-stage cancer

## Abstract

**Background:**

The use of palliative radiotherapy (PRT) is variable in advanced cancer. Little is known about PRT utilization by end-of-life (EOL) cancer patients in Canada. This study examined the PRT utilization rates and factors associated with its use in a cohort of cancer patients who died in British Columbia (BC).

**Methods:**

BC residents with invasive cancer who died between April 1, 2010 and March 31, 2011 were included in the study. Their cancer registry and radiotherapy treatment records were extracted from the BC Cancer Agency information systems and linked for the analysis. The PRT utilization rates by age, sex, primary cancer diagnosis, geographic region, survival time and travel time to the cancer centre were examined. Multivariable logistic regression was used to determine the factors that influenced the PRT utilization rates.

**Results:**

Of the 12,300 decedents in the study 2,669 (21.7%) had received at least one course of PRT in their last year of life. The utilization rates dropped to 5.0% and 2.2% in the last 30 and 14 days of life, respectively. PRT utilization varied across diagnosis and was highest for lung cancer (45.7%) and lowest for colorectal cancer (8.9%). The rates also varied by age, survival time and travel time to the nearest radiotherapy centre. There was a greater odds of receiving PRT for those with primary lung cancer, survival time between 1.5-26 months from diagnosis or living within 2 hours from a cancer centre. The 85+ age group was least likely to receive PRT in their last year of life.

**Conclusions:**

This study found PRT utilization rates of EOL cancer decedents to be variable across the province of BC. Age, diagnosis, survival time and travel time to the nearest radiotherapy centre were found to influence the odds of PRT treatment. Further work is still needed to establish the appropriate PRT utilization rates for the EOL cancer population.

**Electronic supplementary material:**

The online version of this article (doi:10.1186/1472-684X-13-49) contains supplementary material, which is available to authorized users.

## Background

Palliative radiotherapy (PRT) is an established treatment modality in the management of advanced cancer [1]. It is also a potentially effective treatment option for cancer patients at end of life (EOL) to enhance their quality of life when faced with only weeks or months to live [2]. Anecdotally PRT is thought to be commonly used.

Data at the population level on the use of PRT in routine practice is hard to find. One study estimated that approximately 50% of all RT treatments are prescribed with palliative intent [3], but to our knowledge no optimal PRT utilization rates have been established for EOL cancer patients. Several population-based studies on the PRT utilization across major primary cancer sites by EOL cancer patients had been performed in Ontario and Nova Scotia. The overall rates reported in these studies were similar, 22% - 29% [4–6]. While the overall PRT rates reported were similar across the studies, there were significant variations for site-specific rates, e.g. lung cancer rates ranged from 40% to 58% [3, 4]. In addition, many studies found the likelihood of PRT decreased with increased age, lower community median household income, and longer distance from the cancer centre [4–6] and one study [6] found females had lower PRT rates. Aside from these studies, we are not aware of other population-based PRT utilization data or estimated optimal rates for EOL cancer patients in Canada.

Understanding PRT use by EOL cancer patients can provide valuable information for cancer care providers and policy makers in assessing whether these patients have adequate PRT access to improve their quality of life.

This paper describes a retrospective cohort study on PRT utilization rates of EOL cancer decedents in BC. Its objectives were to determine: (a) the PRT utilization rates by age, sex, primary diagnosis, geographic region, survival time and travel time to nearest radiotherapy centre; (b) the proportion of decedents who received at least one course of PRT treatment during the last 30 and 14 days of life; and (c) the decedent, disease and health system related factors associated with receiving PRT in the last year of life.

## Methods

### Study context and population

BC is the third-largest province in Canada with a population of 4.5 million over an area of 947,800 km^2^ [7]. Health care in BC is delivered through one provincial and five regional health authorities. The BC Cancer Agency (BCCA) is part of the provincial authority called the Provincial Health Services Authority and it offers a province-wide, population-based cancer control program. Currently, the BCCA has six cancer centres located across the province to serve BC residents. The province is divided into 16 Health Service Delivery Areas (HSDA). Each of the six cancer centres is responsible for a defined catchment area with a collection of these HSDAs. Note that at the time of this study there were only five cancer centres in operation.

The BC Cancer Registry receives, by law, notification of all initial diagnoses of cancer in BC. The BCCA provides cancer treatment for patients who have been diagnosed with cancer and referred by a physician [8]. The types of treatment provided at the BCCA include RT, chemotherapy and, in Vancouver, surgery. Approximately 60% of the patients who are registered in the BC Cancer Registry will eventually be referred to the BCCA for treatment [9]. Surgical treatments and some chemotherapy treatments are also provided in acute care settings outside of the BCCA. RT treatment is only available through the six cancer centres [10], and all RT treatment records are maintained centrally through the Cancer Agency Information System (CAIS).

### Data sources and study variables

Study Cohort – Patients who died between April 1, 2010 and March 31, 2011 were identified from the Cancer Registry. Those diagnosed with invasive cancer other than non-melanoma skin cancer were included in the study. Those with benign or in situ tumors were excluded. This cohort was used as the denominator for the PRT utilization rates in our study. Note that due to the lack of cause of death data for all study participants, our study cohort consisted of all who died with invasive cancer in a specified time period regardless of cause. The data fields extracted from the Cancer Registry were birthdate, sex, date of death, histology code for primary cancer diagnosis, date and site of diagnosis, and HSDA residence at time of diagnosis. The data fields extracted from CAIS were RT course start date, treatment dose, site and intent. This study was part of a larger EOL care surveillance network project that focused on health service use in the last year of life for identified palliative patients with advanced illness [11]. The variables for this study are defined below.

Course of treatment – The PRT utilization was measured by the course of treatment. For all the treatment sites with exception of Skin and Bone, a course of treatment was uniquely identified by RT start date, end date, and treatment region. For the treatment site of Skin and Bone, every RT record was counted as a new course of treatment regardless of the RT start and end date.

PRT - A course of RT was defined as palliative if there was a palliative intent code provided by the treating radiation oncologist or if the dose of treatment was 30 Gy or less. The dose of 30 Gy was selected as a cut point based on the distribution of RT dose delivered for courses with a palliative intent code in our study cohort (see Additional file 1: Appendix A).

PRT_1Y_ and RT_1Y_ - The proportion of the study cohort that had at least one course of PRT in the last year of life (PRT_**1Y**_) was used to describe utilization of PRT. Similarly, RT_**1Y**_ was used to describe the proportion of the study cohort that had at least one course of RT in the last year of life.

PRT_30d_ and PRT_14d_ – The proportion of the study cohort that had at least one course of PRT in the last 30 (PRT_30d_) and 14 (PRT_14d_) days of life. Last 14 days before death was used by Earle et al. [12] as cut-off for quality of care indicators for cancer care at the end of life; last 30 days was used by Guadagnolo et al. [13] to report on RT utilization for elderly patients in the United States at the end of life.

Primary Diagnosis - Decedents were grouped by their primary cancer diagnosis in ICD-O-3 based on the site and histology codes in their Registry record (see Additional file 2: Appendix B). The primary diagnosis was assigned based on the last known primary cancer diagnosis by the date of diagnosis. If multiple cancer diagnoses from different sites were found on the most recent date of cancer diagnosis, a predefined hierarchy was used to assign the diagnosis based on overall cancer mortality rate: Lung > Breast > Colorectal > Prostate > non-colorectal gastrointestinal (GI) > Blood > Urinary > Female genital > Brain > Cancer-other.

Travel Time – The travel time from a decedent’s residence to the closest cancer centre was measured using distance calculator provided by the British Columbia Automobile Association. It represented the driving time from the centre of the HSDA where the decedent resided when the last primary cancer was diagnosed before he or she died to the closest cancer centre using the postal code of two measurement points. The travel time was grouped into < =2 and >2 hours for each HSDA. The 2-hour cut-off was chosen for each HSDA based on earlier work by Tyldesley et al. [14]. In BC only 4% of the rural regions are within a 2-hour drive of a cancer centre, whereas 46% of the semi-rural regions and 100% of the urban regions are within a 2-hour drive to a cancer centre [14]. The 2-hour distance provided a balanced cut-off that reflects the geographic differences between rural and urban residents in BC.

Survival Time - The time from the last primary cancer diagnosis to death was categorized into three groups of >26 months, 1.5-26 months and <1.5 months. These survival groups had also been used in an earlier study by Lavergne et al. [6] as a predictor of PRT utilization at end of life.

### Statistical analysis

Categorical variables were used to analyze age at death (age < 19, 19–44, 45–64, 65–74, 75–84 or ≥85 years), primary cancer diagnosis (Blood, Brain, Breast, Colorectal, Female Genital, Lung, Melanoma, non-colorectal GI, Prostate, Urinary, or Other), the decedent’s residence in one of 16 HSDAs at time of diagnosis, and survival time from primary cancer diagnosis (<1.5, 1.5-26 or >26 months). Binary variables were used to analyze sex, travel time to the closest cancer centre (≤2 hours/>2 hours), receipt of RT (yes/no) and receipt of PRT (yes/no).

Baseline characteristics were stratified by the receipt of PRT in the last year of life. Logistic regression was used to determine the association between the receipt of PRT and the demographic and clinical factors. Individual factors were modeled first to check for univariable association with the receipt of PRT. Individual variables with a significance level of ≤0.05 were then considered for the multivariable model. Multivariable logistic regression was used to identify the factors associated with the receipt of PRT in the last year of life at a significance level of ≤0.05. Point estimates from the multivariable model were reported as odds ratios (ORs) with the confidence interval (CI) for each OR. All statistical analyses were done with SAS statistical software package (version 9.0; SAS Institute Inc., Cary. NC).

This research was approved by the University of Victoria Ethics Board (#12-262). A waiver from a full ethical review of research involving human participants was received from the University of Victoria Ethics Board due to the fact that this research was limited to second analyses of anonymized data, which cannot identify, or be linked to, the individuals who provided it.

## Results

### Study cohort characteristics

An initial cohort of 13,250 decedents who died between April 1, 2010 and March 31, 2011 were identified from the BC Cancer Registry. The final cohort had 12,300 decedents after excluding 945 with non-melanoma skin cancer and 5 with data quality issues. The majority of the decedents were ≥65 yrs of age. Over half (56.3%) resided in five major geographic regions: Fraser North (10.9%), Fraser South (13.2%), Okanagan (10.5), South Vancouver Island (10.3%) and Vancouver (11.4%). The majority of decedents (76.3%) lived less than 2 hours driving distance from the closest cancer centre. The most common cancer diagnoses were: lung (19.6%), colorectal (12.6%), non-colorectal gastrointestinal (GI) (11.8%), prostate (11.7%) and breast (9.8%). Median survival time was 18.7 months. Close to 15% died within 1.5 months of their last cancer diagnosis while 41.3% died between 1.5 and 26 months from their last cancer diagnosis. The characteristics of the study cohort are summarized in Table 1.

**Table 1:** **Characteristics of study cohort, N = 12,300**

Study cohort	No. of decedents	%	Study cohort	No. of decedents	%
**Age group**			**Residence by Health Service Delivery Area (HSDA)**		
0-18	16	0.1	Central Vancouver Island	1,011	8.2
19-44	226	1.8	East Kootenay	239	1.9
45-64	2,327	18.9	Fraser East	807	6.6
65-74	2,511	20.4	Fraser North	1,346	10.9
75-84	3,613	29.4	Fraser South	1,619	13.2
85+	3,607	29.3	Kootenay Boundary	314	2.6
**Sex**			North Shore/Coast Garibal	820	6.7
Female	5,734	46.6	North Vancouver Island	421	3.4
Male	6,566	53.4	Northeast	124	1.0
**Primary cancer diagnosis**			Northern Interior	371	3.0
Lung	2,406	19.6	Northwest	171	1.4
Breast	1,204	9.8	Okanagan	1,289	10.5
Colorectal	1,551	12.6	Richmond	376	3.1
Prostate	1,438	11.7	South Vancouver Island	1,270	10.3
Non-colorectal GI	1,454	11.8	Thompson Cariboo Shuswap	692	5.6
Blood	938	7.6	Vancouver	1,405	11.4
Urinary	656	5.3	Missing	25	0.2
Female Genital	623	5.1	**Travel time (to closest cancer centre)**		
Brain	255	2.1	≤2 hours	9,364	76.1
Melanoma	265	2.2	>2 hours	2,911	23.7
Other	1,510	12.3	**Radiotherapy (RT)**		
**Survival time from diagnosis**			No RT	9,341	75.9
<1.5 M	1,812	14.7	**RT**	2,959	24.1
1.5-26 M	5,085	41.3	Palliative RT	2,669	21.7
26 M+	5,403	43.9	Nonpalliative RT	290	2.4

### PRT Utilization rates in last year of life

Of the 12,300 decedents in the study cohort, 2,959 (24.1%) were identified as having received at least one course of RT in their last year of life (Table 1). The majority (91.5%) of RT treatments were palliative by our definition. The median time to death following the first course of PRT was 102 days. The overall proportion of decedents who received PRT in the last year was 21.7%.

**Figure 1:**
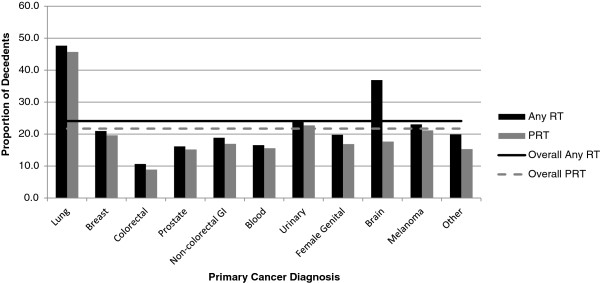
**RT**_**1Y**_ **vs. PRT**_**1Y**_ **rates by primary cancer.** “Palliative” RT is defined as either RT intent = 'palliative' or RT dose < =30Gy.

The RT_1Y_ and PRT_1Y_ rates varied across cancer groups (Figure 1). PRT_1Y_ rates were highest (45.7%) for lung, intermediate (20-30%) for urinary, melanoma and breast, and low (<20%) for non-colorectal GI, prostate, blood, female genital, brain and colorectal. For most cancer groups, the majority of decedents, when treated with RT during their last year of life, received PRT with the exception of those with brain cancer. Just over one-third (36.9%) of brain cancer decedents received RT in their last year of life and less than a fifth (17.6%) received RT deemed palliative by our definition.

**Figure 2:**
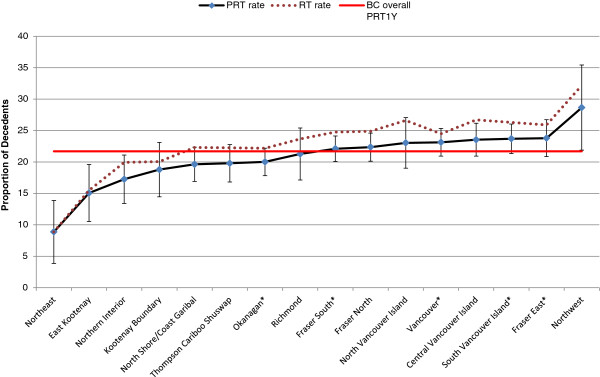
**Regional PRT**_**1Y**_ **rates.** * indicates the region where a cancer center is located. Note: the horizontal line is the average provincial PRT_1Y_ rate and the bars show the PRT_1Y_ rate with a 95% confidence interval for the HSDA.

On average, each decedent received 1.6 PRT courses during their last year of life. The proportion of decedents with at least one course of PRT in last year of life varied across HSDAs (Figure 2). Overall, regions with a cancer centre had higher rates than the provincial average, except for Okanagan, which had below provincial average. PRT_1Y_ rate also was inversely related to travel time. Decedents who resided within two hours to the closest cancer centre received more PRT in the last year of life than those who resided farther from a cancer centre (22.6% vs. 18.8%) (Table 2).

**Table 2:** **Number and percentage of decedents who received PRT**_**1Y**_

Variable	Decedents	Number of decedents who received PRT1Y	% PRT1Y	P value (Chi- square)
Age				
<=44	242	78	32.2	
45-64	2,327	827	35.5	
65-74	2,511	773	30.8	
75-84	3,613	724	20.0	
85+	3,607	267	7.4	<.0001
Sex				
F	5,734	1,269	22.1	
M	6,566	1,400	21.3	0.2774
Primary cancer site				
Blood	938	146	15.6	
Brain	255	45	17.6	
Breast	1,204	236	19.6	
Colorectal	1,551	138	8.9	
Female genital	623	105	16.9	
Lung	2,406	1,099	45.7	
Melanoma	265	56	21.1	
Non-colorectal GI	1,454	246	16.9	
Prostate	1,510	231	15.3	
Urinary	1,438	218	15.2	
Other	656	149	22.7	<.0001
Survival time from diagnosis (Months)				
<1.5	1,812	156	8.6	
1.5-26	5,085	1,727	34.0	
26+	5,403	786	14.5	<.0001
Travel time				
<=2 hours	9,364	2,120	22.6	
>2 hours	2,911	547	18.8	<.0001

### Proportion of PRT in last 30 and 14 days of life

Of the 12,300 decedents in the study, 614 (5.0%) and 268 (2.2%) received PRT in the last 30 and 14 days of life, respectively (Table 3). The proportions that received PRT in the last 30 days varied across cancer groups. The highest proportion was lung cancer at 11.3% while the lowest was colorectal cancer at 1.9%. The remaining cancer groups ranged from melanoma at 4.9% to female genital at 2.4%. In the last 14 days the proportions reduced by 50% or more across all groups. Lung cancer was still the highest at 5.4% but colorectal cancer dropped to 1.0%, while female genital was the lowest at 0.8%. Approximately 3.1% of brain cancer decedents received RT in the last 30 days and 1.6% received in the last 14 days. All RT received by brain cancer decedents in the last month of life were PRT by our definition.

**Table 3:** **PRT rates in last 30 and 14 days of life by primary cancer diagnosis**

Primary cancer diagnosis	Study cohort	Last 30 days				Last 14 days			
		Decedents	% cohort	% PRT	# course	Decedents	% cohort	% PRT	# course
Lung	2,406	272	11.3	24.7	357	129	5.4	11.7	160
Breast	1,204	42	3.5	17.8	54	18	1.5	7.6	20
Colorectal	1,551	30	1.9	21.7	33	16	1.0	11.6	18
Prostate	1,438	43	3.0	19.7	53	20	1.4	9.2	24
Non-colorectal GI	1,454	59	4.1	24.0	69	23	1.6	9.3	24
Blood	938	34	3.6	23.3	50	9	1.0	6.2	17
Urinary	656	36	5.5	24.2	45	11	1.7	7.4	15
Female genital	623	15	2.4	14.3	15	5	0.8	4.8	5
Brain	255	8	3.1	17.8	9	*	*	8.9	*
Melanoma	265	13	4.9	23.2	16	*	*	7.1	*
Other	1,510	62	4.1	26.8	80	29	1.9	12.6	37
Overall	12,300	614	5.0	23.0	781	268	2.2	10.0	328

% cohort: Proportion of decedents received PRT in their last 30/14 days of life.

% PRT: Proportion of decedents who receive PRT courses in last year of life received PRT in their last 30/14 days of life.

#course: Number of PRT courses that decedents received in their last 30/14 days of life.

*Cell is suppressed due to small count.

### Likelihood of receiving PRT in last year of life

The univariable analysis shown in Table 2 showed that patient-related factors such as age at death and time between diagnosis and death, disease-related factors such as primary cancer diagnosis, and health system-related factors such as travel time, were all significantly related to the PRT_1Y_ utilization rates. The age group ≥85 was found to be significantly less likely to receive PRT during their last year of life compared with other age groups. Decedents with primary lung cancer, survival time between 1.5-26 months from diagnosis, or residence in an HSDA area within 2 hours driving distance to a cancer centre were significantly more likely to receive PRT in the last year of life. Sex was not significantly related to PRT_1Y_ rate (*P* = 0.28).

Statistically significant univarable factors were analyzed using multivariable logistic regression. The variations in PRT_1Y_ were presented as adjusted ORs with 95% CIs after controlling for the effects of other variables in Table 4. All factors showed significant independent effects in association with variation in PRT_1Y_. After adjusting for other factors, age at diagnosis was negatively associated with PRT_1Y_ (*p* < 0.0001). Decedents aged 19–44 had 6.7 higher odds of receiving PRT_1Y_ than those greater than 85 years. When compared against lung cancer, decedents with other cancers had significantly smaller odds of receiving PRT_1Y_. For instance, colorectal and brain cancer decedents had 0.1 and 0.2 odds of receiving PRT_1Y_, respectively. When compared with decedents who survived more than 26 months after their last cancer diagnosis, those who survived between 1.5 and 26 months had double the odds of receiving PRT_1Y_, while decedents who survived less than 1.5 months after diagnosis had 0.35 odds of receiving PRT_1Y_. Decedents who travelled less than 2 hours to the closest cancer centre had 1.4 odds of receiving PRT_1Y_ than those who travelled more than 2 hours.

**Table 4:** **Odds ratios describing likelihood of receiving PRT**_**1Y**_

Variable	Adjusted odds ratio	95% CI	
Age ( vs. 85+)			
<19	5.42	1.59	18.50
19-44	6.68	4.83	9.24
45-64	5.64	4.79	6.64
65-74	4.53	3.86	5.32
75-84	2.62	2.24	3.06
Sex			
M (vs. F)	0.97	0.87	1.09
Primary cancer site (vs. Lung)			
Blood	0.24	0.19	0.29
Brain	0.16	0.11	0.23
Breast	0.41	0.34	0.50
Colorectal	0.13	0.11	0.16
Female genital	0.25	0.19	0.32
Melanoma	0.43	0.31	0.60
Non-colorectal GI	0.20	0.17	0.24
Prostate	0.38	0.32	0.47
Urinary	0.41	0.33	0.51
Other	0.23	0.19	0.28
Survival time from diagnosis (Months) ( vs. 26+)			
<1.5	0.35	0.28	0.42
1.5-26	2.01	1.79	2.25
Travel time			
≤2 hours (vs. >2 hours)	1.44	1.28	1.62

## Discussion

### Making sense of PRT utilization

This study showed the overall PRT utilization rate for EOL cancer decedents in the last year of life was 21.7% in BC. This rate was similar to those reported by Lavergne et al. [6] in Nova Scotia (22.5%) for PRT utilization in last 9 months of life and by Huang et al. [4] in Ontario (26.4%) for last 2 years of life.

When compared with Nova Scotia, our rates were lower for breast (19.6 vs. 31.5), melanoma (21.1 vs. 39.5) and prostate (15.2 vs. 27.6) cancer but similar for lung (45.7 vs. 37.3), colorectal (8.9 vs.9.9) and hematologic (15.6 vs.10.3) cancer. Because different variables were used in the Ontario study, PRT rates for only three cancer groups could be compared. Those with breast cancer in Ontario had higher rates of PRT use, (39.4 vs. 19.6), but similar rates for hematopoietic (18.6 vs. 15.6) and lung cancers (39.9 vs. 45.7) despite the difference of time frame reported i.e. 2 year rates in Ontario versus 1 year rates in this study. This study also found brain cancer decedents had the second highest RT utilization rate (36.9%) in the last year of life following after lung cancer; however, only 17.6% of decedents received RT deemed palliative by our definition. This is mainly because brain RT, although palliative by nature, is not likely to be coded as palliative in this data.

In this study, PRT utilization varied by such factors as age, diagnosis, survival time and geographic region. Those younger than 85 years of age, with primary lung cancer, survival time of 1.5-26 months from diagnosis, or residing less than 2 hours from a cancer centre had higher rates of PRT utilization. These results were similar to those identified by Lavergne et al. [6], except for sex; they found being female was associated with declined PRT use. Huang et al. [4] also reported higher likelihood of PRT use among those with younger age and those living near a cancer centre.

Possible reasons for reduced PRT use in older decedents was that they had more severe comorbidities, worse performance status or different treatment preferences that made PRT less suitable [3, 4, 6]. The higher rates for decedents who survived 1.5-26 months may have been because those who had very short survivals may have been too sick to benefit from radiotherapy, as assessed by their oncologists. On the other side of the spectrum, those with longer survivals may have been quite well and asymptomatic, not needing cancer treatment, and may have even died of other causes. The variable rates observed across cancer groups are likely due to difference in symptom frequency/severity or cancer site-specific indications for radiotherapy or other palliative treatments.

Regarding regional differences in PRT use, the lowest PRT rates in this study were in the Northern (i.e. Northeast, Northern Interior), Vancouver Coastal (i.e. North Shore/Coast Garibaldi) and Interior regions (i.e. East Kootenay, Kootenay Boundary, Thompson, Okanagan). One likely reason for the low rates in the Northern region may have been that there was no cancer centre in the region at the time of this study, and decedents were generally referred to the Vancouver centre, which could have been many hours flight from many parts of the regions. Another was that North East and East Kootenay HSDAs are close to the neighbouring province of Alberta. It is possible that some decedents were referred to the radiotherapy centres in Alberta, which would not be captured in our data [15]. These findings were consistent with other studies that showed the detrimental effect of increased travel time on reduced PRT use existed previously, affecting patients as early as 1986 [15, 16].

One exception to the regional variation in PRT use was the Northwest HSDA in the Northern region which had the highest rate (28.6) in the province. This could relate to a high percentage of First Nation population residing in the region, whose travel may have been more likely to be paid for. Another was the Interior Okanagan HSDA that had rates (20.0) below the provincial average despite having a cancer centre in the region. One reason could be that 20-40% of the decedents in that HSDA were more than 2 hours drive away from the cancer centre. Another reason could be that pre-existing long wait lists for radiotherapy may have been a significant disincentive to refer patients for PRT, or patients may have died or been treated with other palliative measures prior to PRT becoming available to them.

Because of the methodology of this study, we were unable to examine other relevant patient, service and system related factors that may influence PRT utilization, such as functional status and symptoms [13, 17], socioeconomic status [4–6, 13], hospice care [4], nursing home residency [6], PRT consultation [5, 6], hospital affiliation [13], wait times [18, 19], referral to a formal palliative care program [20, 21], and physicians’ knowledge of PRT and how active they were involved in palliative care [18]. The availability of formal Palliative Care programs and hospice care varies greatly among communities in British Columbia. Further work needs to be done to link the availability of these services to outcomes.

### Appropriate PRT use in EOl cancer care

In this study, the proportion of EOL cancer decedents that received at least one course of PRT in the last 30 and 14 days of life were 5.0% and 2.2%, respectively. Guadagnolo et al. [13] studied 15,287 SEER-Medicare decedents (≥65 yrs) who died from malignant diseases of lung, breast, prostate, colorectal, and pancreas from 2000 to 2007. In that study, 7.6% of the decedents received PRT within their last 30 days of life. The lower rate observed in our study may be partially explained by the differences in defining the study cohort as well as the reimbursement systems used in different health care systems. Despite the underutilization of PRT in end-of-life care in Guadagnolo et al. study [13] and the others [2, 17, 22], due to the relevance of nonclinical factors found with the use of PRT in the last month of life, we would argue that the lower rate we found in our study might indicate the accuracy of physician predictions on prognosis and thus ‘appropriate’ palliative care, especially with respect to geographic access. Lower rates are considered appropriate, as good palliative care for a patient who lives far away is through use of medication and avoidance of a long trip that might hasten their death and negatively impact their overall quality of life.

Some studies reported “optimal” PRT utilization rates based on empirical findings or model estimates. For instance, Huang et al. [4] identified a subgroup of cancer decedents in Ontario who had more optimal PRT (2 year) access rate of 57.8%, which was above the average of 26.4% in their study population. This subgroup was made up of individuals who were less than 70 years of age, residents of medium and high income communities, diagnosed in a hospital with a cancer centre, and residing near cancer centres with better PRT access. Based on these findings, Huang et al. concluded that PRT was underutilized in Ontario. In our study, due to the lack of neighbourhood income data, we were not able to construct a comparable cohort to compare the ‘optimal’ PRT rate for BC EOL cancer decedents. It is impossible to truly asses if PRTs rates found in this study are “appropriate” or “inappropriate”, given the lack of individual decedent data about pain and symptom prevalence/severity, decedent functional status, and decedent treatment preferences. Nevertheless, the fact that PRT rates in our study varied by non-clinical factors such as age, travel time and geographic region does suggest underutilization of PRT in a subgroup of population with suboptimal access.

With the increased emphasis on monitoring EOL care, further work is needed to assess appropriateness of PRT rate for EOL cancer decedents in BC, and to develop benchmark rates that can be practically assessed in different geographic regions. Development of these benchmark rates may require richer data collected at the point of care as well as rigorous/robust methodology based on a sound scientific footing. Both aspects remain challenging in the current palliative/EOL care research but desire resolution in the future.

### Study limitations and implications

This study has three limitations. First, the cause of death for decedents was not available at the time of data collection. Therefore the cohort consisted of decedents who died with rather than *of* cancer, which could under-estimate PRT rates. Second, this study used data collected for administrative purposes. Such clinical factors as symptom assessment and patient preferences at end of life were not available, which could provide reasons for PRT use. Third, PRT practice in BC may not be generalizable outside of BC, as practice varies by country and training, location and type of practice, experience and reimbursement.

To the best of our knowledge, this population-based study is the first of its kind providing data on the use of PRT for the end of life cancer population in BC. It is our hope that this information could be used by local cancer care providers and health care administrators for cancer care planning as well as researchers conducting end of life care research. We believe that the findings from this study have shed light on the issues of suboptimal PRT treatment received by a subgroup of the BC cancer population due to restricted geographic access. Identifying the subgroup who might be receiving suboptimal care at the end of life will help service planners target this population for quality improvement. Even though it is not an intention of this research to identify optimal PRT rates, findings from this study raises important questions and provides the base PRT utilization rate at the population level for future research. Lastly, this study will add province-specific data of BC to existing data available in other Canadian provinces on the subject of PRT utilization for palliation. This will increase the availability of data on provincial comparisons for future end of life research.

## Conclusions

This study examined PRT utilization by cancer decedents in their last year of life in BC. The overall PRT rate was 21.7%. The likelihood of receiving PRT EOL cancer decedents was higher for those less than 85 years of age, with primary lung cancer, survival time of 1.5-26 months from diagnosis, or living less than 2 hours from a cancer centre. The proportion of EOL cancer decedents who had received at least one course of PRT in the last 30 and 14 days of life were 5.0% and 2.2%, respectively. It is not clear whether these rates are considered optimal as there are no established PRT utilization rates for EOL cancer decedents at present.

## Electronic supplementary material

Additional file 1: Appendix A: Distribution of Doses for RT courses with “Palliative Intent” Code. Among all the RT courses (4776) prescribed to the study cohort during the last year of life, 88.4% (4221) of the courses had a “palliative intent” code. The majority (94.5%) of them were prescribed with dose < =30 Gy. (PPTX 86 KB)

Additional file 2: Appendix B: Definitions of Primary Cancer Diagnosis. This file provides the details about the cancer diagnosis (ICD-O-3) codes used in this study. (PDF 212 KB)

## Abbreviations

PRT: Palliative radiotherapy
RT: Radiotherapy
EOL: End of life
BCCA: BC cancer agency
HSDA: Health service delivery area
CAIS: Cancer agency information system
OR: Odds ratio
CI: Confidence interval.
