# Supplementary figures and images for: Palliative radiotherapy utilization for cancer patients at end of life in British Columbia: retrospective cohort study
Source: BMC Palliat Care. 2014 Nov 18;13:49. doi: 10.1186/1472-684X-13-49 (PMC4240806; doi:10.1186/1472-684X-13-49)

## Slide 1
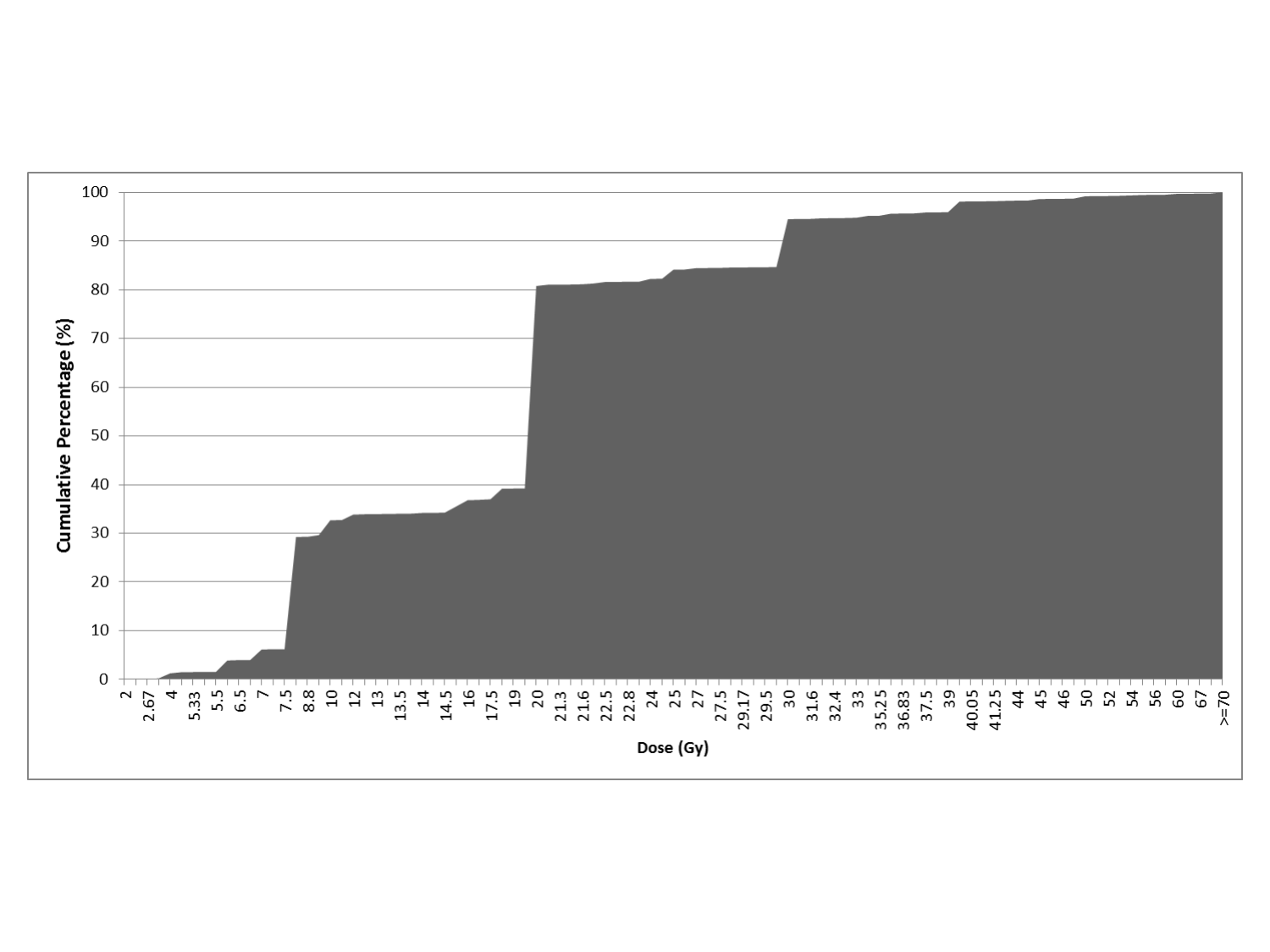

Supplement: Supplementary file 1 — Additional file 1: Appendix A: Distribution of Doses for RT courses with “Palliative Intent” Code. Among all the RT courses (4776) prescribed to the study cohort during the last year of life, 88.4% (4221) of the courses had a “palliative intent” code. The majority (94.5%) of them were prescribed with dose < =30 Gy. (PPTX 86 KB) [file 12904_2014_230_MOESM1_ESM.pptx]
