# Supplementary material for: Palliative radiotherapy utilization for cancer patients at end of life in British Columbia: retrospective cohort study
Source: BMC Palliat Care. 2014 Nov 18;13:49. doi: 10.1186/1472-684X-13-49 (PMC4240806; doi:10.1186/1472-684X-13-49)
Supplement: Supplementary file 2 — Additional file 2: Appendix B: Definitions of Primary Cancer Diagnosis. This file provides the details about the cancer diagnosis (ICD-O-3) codes used in this study. (PDF 212 KB) [file 12904_2014_230_MOESM2_ESM.pdf]

Appendix B: Definitions of Primary Cancer Diagnosis

Primary Cancer Site	ICD-O-3 Site	ICD-O-3 Site Description	Histology Code Exclusion
**Lung**	**C34**		excluding histology code 9590-9989, 9050-9055, 9140
	C340	Malignant neoplasm of main bronchus	
	C341	Malignant neoplasm upper lobe, bronchus or lung	
	C343	Malignant neoplasm lower lobe, bronchus or lung	
	C348	Overlapping malignant lesion of bronchus and lung	
	C349	Malignant neoplasm bronchus or lung unspecified	
**Colorectal**	**C18-C20**		
	C18	Malignant neoplasm of colon	
	C19	Malignant neoplasm of recto sigmoid junction	
	C20	Malignant neoplasm of rectum	
**Prostate**	**C61**	**Malignant neoplasm of prostate**	
**Breast**	**C50**		
	C500	Malignant neoplasm of nipple and areola	
	C501	Malignant neoplasm of central portion of breast	
	C502	Malignant neoplasm of upper-inner quadrant of breast	
	C503	Malignant neoplasm of lower-inner quadrant of breast	
	C504	Malignant neoplasm of upper-outer quadrant of breast	
	C505	Malignant neoplasm of lower-outer quadrant of breast	
	C506	Malignant neoplasm of axillary tail of breast	
	C508	Overlapping malignant lesion of breast	
	C509	Malignant neoplasm breast part unspecified	
**Brain**	**C70-C72**		
	C70	Malignant neoplasm of meninges	
	C71	Malignant neoplasm of brain	
	C72	Malignant neoplasm of spinal cord, cranial nerves and other parts of central nervous system	
**Blood**			
Hodgkin Lymphoma		All primary sites with histology codes 9650-9667	
Non-Hodgkin Lymphoma		All primary sites with histology codes 9590-9596, 9670-9719, 9727-9729<br>histology codes 9823, all sites except C42.0, C42.1, C42.4<br>histology codes 9827, all sites except C42.0, C42.1, C42.4	
Multiple Myeloma		All primary sites with histology codes 9731, 9732, 9734	
Leukemia		All primary sites with histology codes 9733, 9742, 9800-9801, 9805, 9820, 9826, 9831-9837, 9840, 9860-9861, 9863, 9866-9867, 9870-9876, 9891, 9895-9897, 9910, 9920, 9930-9931, 9940, 9945-9946, 9948, 9963-9964<br>For sites C42.0, C42.1, C42.4, histology codes 9823 and 9827	

Primary Cancer Site	ICD-O-3 Site	ICD-O-3 Site Description	Histology Code Exclusion
**Other Digestive (excluding colorectal)**	**C15-C17, C21-C26**		excluding histology code 9590-9989, 9050-9055, 9140
Esophagus	C15	Malignant neoplasm of oesophagus	
Stomach	C16	Malignant neoplasm of stomach	
Small intestine	C17	Malignant neoplasm of small intestine	
Anus and anal canal	C21	Malignant neoplasm of anus and anal canal	
liver	C22	Malignant neoplasm of liver and intrahepatic bile ducts	
Gallbladder	C23	Malignant neoplasm of gallbladder	
Other and unspecified parts of biliary tract	C24	Malignant neoplasm of other and unspecified parts of biliary tract	
Pancreas	C25	Malignant neoplasm of pancreas	
Intestinal tract	C26	Malignant neoplasm of other and ill-defined digestive organs	
**Skin - Melanoma**	**C44 and histology codes 8720-8790**		
	C44	Other malignant neoplasms of skin	
**Urinary**	**C64-C68**		excluding histology code 9590-9989, 9050-9055, 9140
Kidney	C64	Malignant neoplasm of kidney, except renal pelvis	
Kidney - renal pelvis	C65	Malignant neoplasm of renal pelvis	
Ureter	C66	Malignant neoplasm of ureter	
Bladder	C67	Malignant neoplasm of bladder	
Other and unspecified urinary organs	C68	Malignant neoplasm of other and unspecified urinary organs	
**Female genital**	**C51-C58**		
Vulva	C51	Malignant neoplasm of vulva	
Vagina	C52	Malignant neoplasm of vagina	
Cervix	C53	Malignant neoplasm of cervix uteri	
Body of Uterus	C54	Malignant neoplasm of corpus uteri	
	C55	Malignant neoplasm of uterus, part unspecified	
Ovary	C56	Malignant neoplasm of ovary	
	C57	Malignant neoplasm of other and unspecified female genital organs	
	C58	Malignant neoplasm of placenta	
